# Etymologia: Rhabdomyolysis

**DOI:** 10.3201/eid2607.ET2607

**Published:** 2020-07

**Authors:** Ronnie Henry

**Keywords:** rhabdomyolysis, breakdown, sketal muscle, striated muscle, myoglobin, kidney failure, bible, Book of Numbers

## Rhabdomyolysis [rabʺdo-mi-olʹə-sis]

From the Greek *rhabdos* (“rod”) + *mus* (“muscle”) + *lusis* (“loosening”), rhabdomyolysis refers to the rapid breakdown of skeletal (striated) muscle, releasing myoglobin into the blood, which can lead to kidney failure. In the Book of Numbers in the Bible, the Israelites grew tired of eating manna. They demanded that God send them meat. God, angry at their insolence, sent them quail but then strikes those who ate the meat with a plague (Numbers 11:31–35). This may have been an early account of rhabdomyolysis, since migrating quail (Figure) eat large amounts of hemlock, a known cause of rhabdomyolysis.

**Figure:**
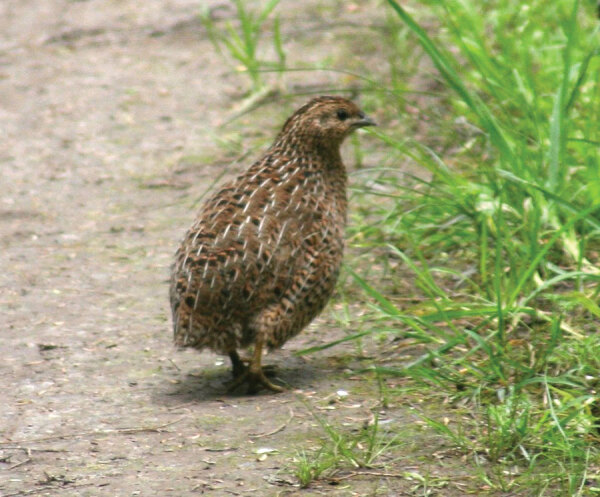
Brown quail (*Coturnix ypsilophora*) by Duncan Wright - Own work, CC BY-SA 3.0, https://commons.wikimedia.org/w/index.php?curid=2998176
